# Rehabilitation assessment of upper limb motor function in stroke patients based on semi-quantitative information

**DOI:** 10.3389/frobt.2026.1830732

**Published:** 2026-05-28

**Authors:** Jiansheng Ding, Wenchao Sun, Tiecheng Ji, Manlin Chen, Dawei Jiang

**Affiliations:** 1 Applied Technology College, Changchun University of Technology, Changchun, China; 2 Computer Science and Engineering, Changchun University of Technology, Changchun, China; 3 Rehabilitation Department, Jilin Electric Power Hospital, Changchun, China

**Keywords:** belief rule base, evidential reasoning, semi-quantitative information, upper limb horizontal rehabilitation robot, upper limb rehabilitation evaluation

## Abstract

During stroke rehabilitation, the recovery of upper-limb motor function is a primary therapeutic objective. Accurate rehabilitation assessment is essential for the scientific design and implementation of individualized training programs. However, current clinical practice predominantly relies on scale-based manual evaluations, which depend heavily on clinicians’ subjective judgment and may compromise objectivity and accuracy. To overcome this limitation, this study develops a novel horizontal upper-limb rehabilitation robot for quantifying patient movement data and proposes an upper-limb rehabilitation assessment model based on semi-quantitative information. The model integrates quantitative data collected by the robot with expert knowledge, employing a BRB algorithm to dynamically and objectively evaluate patients’ rehabilitation status. To validate the accuracy and effectiveness of the proposed method, BP and SVM neural networks are adopted for comparative models. Comparative analysis demonstrates that the semi-quantitative information-based BRB model achieves significantly higher accuracy than the alternative models. This approach not only enables rapid and precise monitoring of patients’ rehabilitation progress but also provides a robust foundation for the scientific formulation of individualized rehabilitation training plans.

## 1 Introduction

Stroke, also known as a cerebrovascular accident, is an acute neurological disorder caused by the sudden rupture or occlusion of cerebral blood vessels, resulting in brain tissue damage (Tsao et al., 2023). Patients with stroke commonly present with unilateral or bilateral limb weakness, speech impairment, and cognitive dysfunction, and in severe cases, the condition may be life-threatening (Langhorne et al., 2011). Stroke not only severely affects patients’ quality of life but also imposes substantial physical and psychological burdens, with upper-limb motor dysfunction having a particularly significant impact on activities of daily living. Early standardized treatment can effectively reduce stroke-related mortality and disability. In current clinical practice, rehabilitation therapists typically assist patients with early-stage rehabilitation interventions. Scientific rehabilitation training can partially restore upper-limb motor function. With technological advances and increasing clinical demand, rehabilitation robots have gradually been introduced into clinical settings. Under the supervision of therapists, individualized rehabilitation programs are designed, therapeutic movements are assisted, and subsequent training plans are adjusted based on rehabilitation assessments (Maciejasz et al., 2014).

Upper-limb rehabilitation is essential for patients with motor impairments resulting from neurological diseases or trauma, and accurate assessment is a prerequisite for effective rehabilitation training. In clinical practice, therapists often rely on individual assessment scales to conduct quantitative or qualitative evaluations and subsequently formulate rehabilitation plans to achieve optimal therapeutic outcomes. Commonly used upper-limb assessment scales include the Brunnstrom Stage, Ueda Assessment Scale, Fugl-Meyer Assessment (FMA), and Modified Ashworth Scale (Gladstone et al., 2002). However, such assessment methods heavily depend on the subjective expertise of therapists and are time-consuming, which imposes significant limitations on both clinicians and patients (Schweighofer et al., 2012). Therefore, introducing intelligent systems for upper-limb rehabilitation assessment has substantial practical significance.

Currently, most rehabilitation robots primarily assist therapists in delivering rehabilitation training, while their capability for objective evaluation of patients’ rehabilitation outcomes remains limited (Rahman et al., 2023; Zhang et al., 2023). This limitation highlights the necessity of integrating rehabilitation robots with artificial intelligence and machine learning techniques to enable quantitative analysis of patient recovery. Consequently, the development of computer-assisted rehabilitation assessment systems has become an active research focus, with numerous studies exploring related approaches (Kumar et al., 2017; Shestakov and Keller, 2021; McInerney and Nieuwenhuis, 2009). For example, Miao et al. designed a smartphone-based intelligent system that utilizes built-in multimodal sensors to capture motion data and evaluates rehabilitation performance using a DTW–KNN hybrid algorithm (Miao et al., 2021). Gui et al. developed an upper-limb rehabilitation training and assessment system based on EEG and EMG signals, analyzing brain–muscle synchronization during rehabilitation and resting states to investigate variations in brain network parameters across recovery stages (Gui et al., 2019). Kim proposed a method for assessing elbow spasticity and applied machine learning algorithms to classify spasticity severity (Kim et al., 2020). Le et al. developed a deep learning-based autonomous limb assessment system enabling online consultation between patients and therapists (Ding et al., 2023). Zhang et al. proposed a desktop rehabilitation robot-based evaluation system capable of quantitatively assessing upper-limb motor function and tracking patient progress (Zhang et al., 2022). Yan et al. designed a portable intelligent system for digital stroke rehabilitation assessment to support both diagnosis and monitoring (Yan et al., 2024). Yu et al. developed a wearable sensor-based evaluation system using two accelerometers and seven bend sensors to monitor upper-limb, wrist, and finger function, demonstrating good correlation with FMA scores (Yu et al., 2016).

From a research paradigm perspective, upper-limb rehabilitation assessment methods can be categorized into data-driven, knowledge-driven, and semi-quantitative hybrid approaches. Data-driven methods, represented by machine learning models such as SVM and BP neural networks, rely on motion data for automated assessment and show strong performance in nonlinear fitting and pattern recognition. However, they typically require large training datasets, have limited interpretability, and struggle to incorporate clinical expertise. Knowledge-driven methods are based on rule systems that encode clinical knowledge and offer interpretability, but they have limited capability in handling complex continuous data and relatively low flexibility. In contrast, semi-quantitative hybrid methods integrate quantitative data with qualitative knowledge, making them more suitable for the inherent fuzziness, uncertainty, and expert dependence in rehabilitation assessment. In practice, most device-based systems rely purely on quantitative indicators derived from motion data without incorporating clinicians’ subjective judgments. However, clinical evaluation requires comprehensive decision-making based on patient-specific conditions, where expert experience is indispensable (Kelliher et al., 2020; Meng et al., 2015). Therefore, an ideal rehabilitation assessment system should integrate quantitative measurements with qualitative expert knowledge to achieve more comprehensive, reliable, and clinically meaningful results.

The belief rule base (BRB) is a modeling approach capable of effectively utilizing various forms of semi-quantitative information to address complex decision-making problems. The concept of BRB was first proposed by Professor Jian-Bo Yang (2006) at the University of Manchester in 2006. Subsequently, Zhijie Zhou further enriched and developed the BRB framework, systematically proposing structural optimization and parameter learning strategies, and advancing BRB research (Chang et al., 2018; Cao et al., 2021; Chen et al., 2021). As an advanced method in complex system modeling, BRB can effectively integrate semi-quantitative information and represent multi-source uncertain knowledge. In recent years, BRB has been gradually introduced into upper-limb rehabilitation assessment research. Jiang et al. developed an upper-limb rehabilitation evaluation model based on BRB by incorporating multiple features related to upper-limb motor performance into the BRB inference framework, and employed evidential reasoning (ER) to achieve rule aggregation and parameter optimization, thereby enabling a comprehensive assessment of patients’ upper-limb rehabilitation status (Jiang et al., 2024). On this basis, Li et al. further established a BRB-based evaluation model for upper-limb motor function assessment in stroke patients and conducted a comparative analysis between the model inference results and the MSS scale scores. The experimental results demonstrated good consistency between the BRB model outputs and the MSS scale, validating the feasibility and effectiveness of BRB for upper-limb rehabilitation function assessment (Li et al., 2024). However, existing BRB-based rehabilitation assessment methods are mostly constructed using general motion features or offline data, and lack close integration with the actual motion patterns of horizontal upper-limb rehabilitation robots. Most studies focus on joint angles or simple kinematic indicators, which cannot fully describe movement outcome, process smoothness, and active force output simultaneously. Furthermore, few studies have realized a closed-loop system that combines robot data collection, feature extraction, and semi-quantitative assessment in a unified framework.

Therefore, this study developed a two-degree-of-freedom horizontal upper-limb rehabilitation robot for rehabilitation training and motion data acquisition in stroke patients. Unlike traditional clinical scales that primarily assess upper-limb function based on muscle strength and joint angles, TO was adopted as a key evaluation feature instead of joint angles. In addition, MS and HPF were incorporated to comprehensively characterize motor performance from the perspectives of movement outcome, motion process, and dynamic capability. Based on these features, a semi-quantitative rehabilitation assessment model was constructed by integrating expert knowledge, and the DE algorithm was employed to optimize the model parameters, thereby reducing subjectivity and improving the objectivity and accuracy of rehabilitation assessment. BP neural network and SVM were used as baseline models to validate the accuracy and generalization ability of the proposed method. This study aims to address the limitations of strong subjectivity in clinical assessment and the incompleteness of robot-based evaluation, providing theoretical and technical support for precise and individualized rehabilitation.

## 2 Rehabilitation robot design

### 2.1 Structure of rehabilitation robot

**FIGURE 1:**
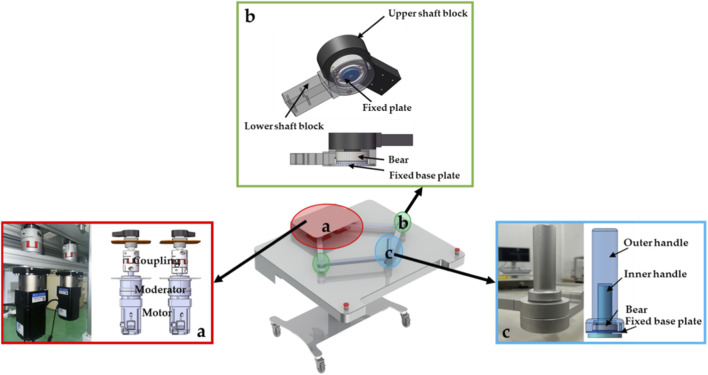
Three-dimensional model of upper limb rehabilitation robot based on five-bar mechanism: **(a)** Motor connection structure diagram, and **(b)** 3D model of connecting rod coupling part, and **(c)** 3D model of handle.

Parallel robots are ideal tools for rehabilitation training. As shown in Figure 1, the 5-bar upper-limb rehabilitation robot is primarily an end-effector-guided device capable of performing planar training. The robot consists of four links of equal length connected to the end-effector in a parallel configuration. This structure enables the robot to execute various rehabilitation trajectories within the horizontal plane, accurately replicating movements commonly performed in daily life. Moreover, the robot is equipped with a lifting mechanism that allows flexible adjustment of the desktop height, enabling patients to perform rehabilitation exercises at an appropriate level according to their individual needs. The overall platform height ranges from 700 to 1200 mm. In terms of actuation, the links are driven by two servo motors. To increase output torque and reduce output speed, each motor is coupled with a planetary gear reducer.

Figure 1 illustrates that the 5-bar upper-limb rehabilitation robot is designed with careful consideration of mechanical connections and patient interaction. The links are connected using fixed plates and bearings, which securely join the upper and lower connecting blocks, allowing relative rotation while preventing vertical separation (Figure 1a). At the end-effector, the inner-axis handle is rigidly attached to the link, while the outer-axis handle is mounted on a bearing through a fixed bottom plate and connected to the inner handle via a stepped shaft, enabling independent rotation to accommodate wrist movements (Figure 1b). An elbow support can be incorporated to provide additional assistance to the affected limb within the horizontal plane, thereby reducing gravitational load during training. The servo motors and planetary gear reducers are installed on the profile frame using fixed plates, and torque is transmitted to the links via couplings to drive the patient’s movements, thereby facilitating precise and controlled rehabilitation exercises (Figure 1c).

### 2.2 Forward kinematics of rehabilitation robot

**FIGURE 2:**
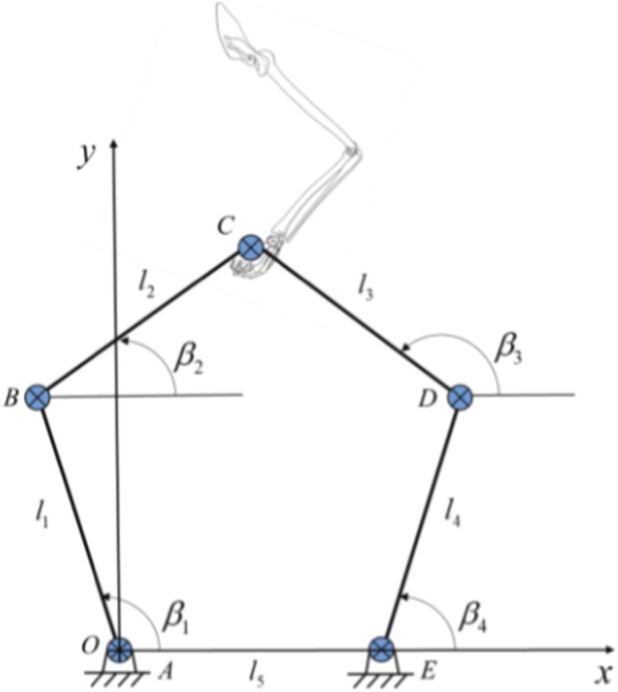
Kinematic model of the 5-bar upper-limb rehabilitation robot.

The kinematic schematic of the 5-bar upper limb rehabilitation robot is shown in Figure 2. The mechanism has two degrees of freedom, with the line segment OE serving as the fixed base. Point A, where link $l_{1}$ connects to the fixed base, is defined as the origin of the global coordinate system, with the x-axis aligned along the frame and passing through the rotation center of one of the actuators. The revolute joints at points A and E are driven by two servo motors equipped with absolute encoders, allowing real-time measurement of the phase angles $\beta_{1}$ and $\beta_{4}$ of links $l_{1}$ and $l_{4}$ .

As shown in Figure 2, by projecting the left and right parts of the 5-bar linkage onto the x and y-axes, the position equations of points B and D can be obtained, as expressed in Equation 1.

$$\left\{\begin{array}{ll} x_{B}=l_{1}\cos\beta_{1}, & y_{B}=l_{1}\sin\beta_{1}\\ x_{D}=l_{4}\cos\beta_{4}+l_{5}, & y_{D}=l_{4}\sin\beta_{4} \end{array}\right.$$ (1)

Accordingly, the position equation of the end-effector point C can be derived:

$$\left\{\begin{array}{l} x_{C}=x_{B}+l_{2}\cos\beta_{2}=x_{D}+l_{3}\cos\beta_{3}\\ y_{C}=y_{B}+l_{2}\sin\beta_{2}=y_{D}+l_{3}\sin\beta_{3} \end{array}\right.$$ (2)

By rearranging Equation 2 to collect the terms containing $\beta_{3\;}$ on one side and applying trigonometric identities, both sides of the equation are squared and summed. After simplification, Equation 3 is obtained:

$$l_{3}^{2}=l_{BD}^{2}+2l_{2}\left(x_{B}-x_{D}\right)\cos\beta_{2}+2l_{2}\left(y_{B}-y_{D}\right){\sin\beta_{2}+l_{2}}^{2}$$ (3)

Finally, the linear trigonometric equation with respect to $\beta_{2}$ is obtained:

$$\beta_{2}=2\arctan\left(\frac{B_{0}\pm \sqrt{A_{0}^{2}+B_{0}^{2}-C_{0}^{2}}}{A_{0}+C_{0}}\right)$$ (4)

where,

$$\begin{array}{ll} & A_{0}=2l_{2}\left(x_{D}-x_{B}\right)\\ & B_{0}=2l_{2}\left(y_{D}-y_{B}\right)\\ & C_{0}=l_{2}^{2}+l_{BD}^{2}-l_{3}^{2}\\ & l_{BD}=\sqrt{{\left(x_{D}-x_{B}\right)}^{2}+{\left(y_{D}-y_{B}\right)}^{2}} \end{array}$$

Substituting $\beta_{2}$ into Equation 2, the coordinates $x_{C}$ and $y_{C}$ can be obtained, and

$$\beta_{3}=\arctan\left(y_{c}-\frac{y_{D}}{x_{c}}-x_{D}\right)$$

in Equation 4, the symbols “*+*” and “*−*” correspond to the upper and lower assembly configurations of point C, respectively. To avoid singular configurations of the upper-limb rehabilitation robot, the “*+*” solution is selected in this study.

**FIGURE 3:**
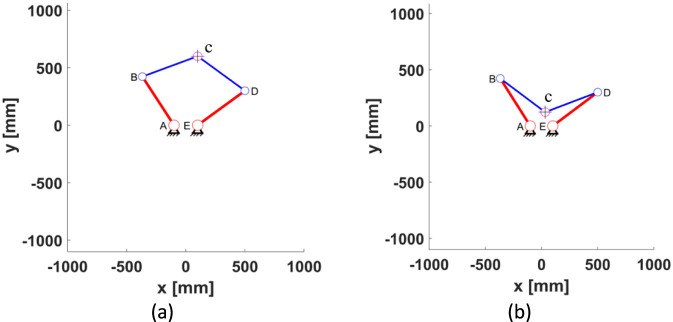
Two configurations of the robot when $\beta_{1}=123^{0}$ and $\beta_{4}=37^{0}$ : **(a)** upper configuration and **(b)** lower configuration.

Figure 3 illustrates the structural parameters of the upper-limb rehabilitation robot based on a 5-bar mechanism are given as $l_{1}=l_{2}=l_{3}=l_{4}=500mm$ and $l_{5}=200mm.$ When the input angles are $\beta_{1}=123^{0}$ and $\beta_{4}=37^{0}$ *,* $\beta_{2}$ is obtained by solving (Equation 4), and then substituted into Equation 2 to determine $x_{C},y_{C}$ and $\beta_{3}$ .

### 2.3 Workspace of rehabilitation robot

**FIGURE 4:**
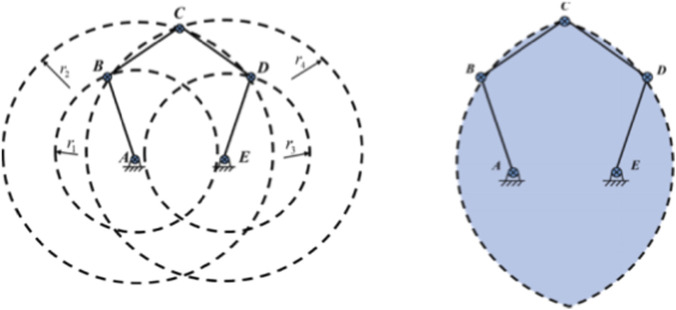
Schematic diagram of the 5-bar motion domain.

To determine whether the designed robot can meet the rehabilitation requirements of patients, it is necessary to investigate its workspace. As shown in Figure 4, the region inside the circle with radius $r_{1}$ is defined as $R_{1}$ , while the region outside this circle is denoted as $S_{1}.$ The region inside the circle with radius $r_{2}$ is defined as $S_{2}.$ The region inside the circle with radius $r_{3}$ is defined as $R_{3}$ , whereas the region outside this circle is denoted as $S_{3}$ .The region inside the circle with radius $r_{4}$ is defined as $S_{4}.$ Accordingly, the theoretical reachable workspace of point C in the 5-bar mechanism can be expressed as:

$$S_{1}\cap S_{2}\cap S_{3}\cap S_{4}$$

Figure 4 illustrates the interactions among the four circles require further analysis to determine their intersecting regions. In this study, the link lengths of the 5-bar upper-limb rehabilitation robot were set as ${\;l}_{1}=l_{2}=l_{3}=l_{4}=500mm$ , the base link $l_{5\;}=200mm$ . By calculating with Equation 5, the resulting parameters were calculated as $r_{1}=0$ , $r_{2}=1000mm$ , $r_{3}=0$ , and $r_{4}=1000mm$ .

$$\left\{\begin{array}{l} r_{1}=\left|l_{2}-l_{1}\right|\\ r_{2}=l_{2}+l_{1}\\ r_{3}=\left|l_{3}-l_{4}\right|\\ r_{4}=l_{3}+l_{4} \end{array}\right.$$ (5)

**FIGURE 5:**
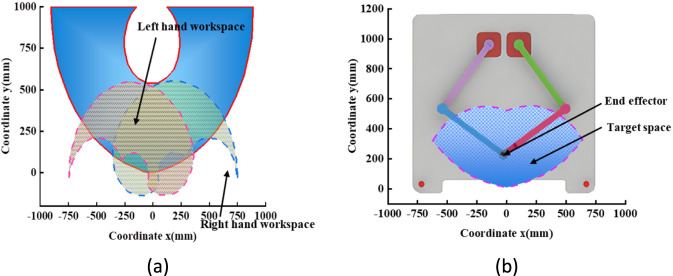
Schematic diagram of the workspace distribution of the 5-bar mechanism: **(a)** independent workspace of individual links, and **(b)** cooperative workspace of the mechanism.

In practical applications, the usable workspace should exclude singular configurations and consider structural constraints as well as inter-link interference. Accordingly, the motion ranges of the actuated links are restricted to 0^∘^–180^∘^. By combining the mechanism design parameters with inter-link interference analysis, the actual usable workspace of the upper-limb rehabilitation robot determined, as illustrated in Figure 5.

### 2.4 Singularity analysis of rehabilitation robot

Singularities refer to specific configurations in which the motion or force transmission of a mechanism becomes indeterminate or uncontrollable. Near such configurations, the kinematic and dynamic performance of the mechanism deteriorates significantly; therefore, singularities should be avoided or maintained at a safe distance during operation. For the proposed device, singular configurations mainly occur in two forms: serial singularities and parallel singularities.

**FIGURE 6:**
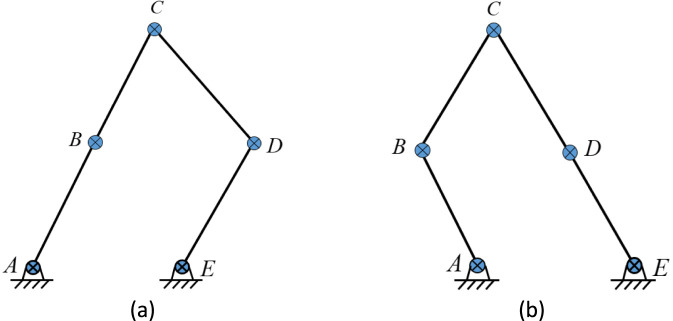
Serial singularities: **(a)** $l_{1}$ collinear with $l_{2}$ and **(b)** link $l_{3}$ collinear with link $l_{4}$ .

Figure 6 illustrates that a serial singularity occurs when link ${\;l}_{1}$ and link ${\;l}_{2}$ are collinear or link ${\;l}_{3}$ and link ${\;l}_{2}$ are collinear. Accordingly, two such serial singular configurations exist.

**FIGURE 7:**
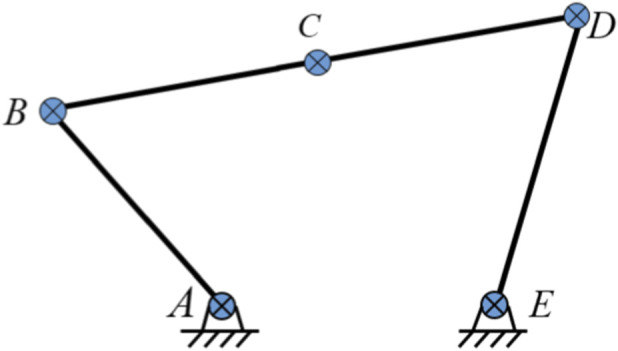
Parallel singularities.

Figure 7 illustrates that a parallel singularity occurs when link $l_{2}$ and link $l_{3}$ become collinear. In total, two such singular configurations exist. At these configurations, the two-degree-of-freedom 5-bar mechanism loses mobility, and the linkage cannot be driven regardless of the magnitude of the applied input force.

For the two degree-of-freedom 5-bar mechanism, singular configurations primarily occur at parallel singularities due to its inherent kinematic characteristics. However, by appropriately coordinating the two input motions, the mechanism can effectively avoid these singular configurations.

### 2.5 Experiment of rehabilitation robot

**FIGURE 8:**
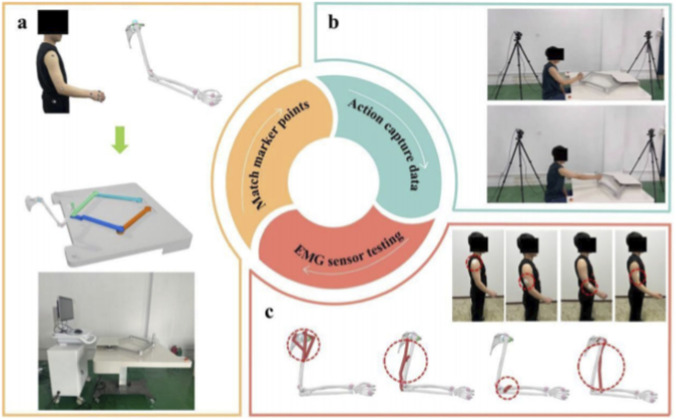
Upper limb feature data acquisition map: **(a)** location of subject marker points, and **(b)** ocation of marker points in the model, and **(c)** motion capture process.

In rehabilitation,the primary objective of robotic systems is to assist patients in regaining the ability to perform activities of daily living. Figure 8 depicts a 5-bar upper-limb rehabilitation robot, designed according to the structural characteristics of a closed-loop 5-bar mechanism. Through motor actuation and linkage transmission, the robot achieves precise control of upper-limb movements. Based on kinematic analysis, the system calculates the real-time position of the end effector (point C), enabling multi-dimensional motion control in both active and passive training modes. The robot can accurately reproduce various trajectory patterns, including linear, curved (e.g., circular and elliptical), and polygonal (e.g., triangular and rectangular) trajectories. Each trajectory type corresponds to distinct coordination patterns of the shoulder, elbow, and wrist joints, allowing the system to accommodate individualized rehabilitation requirements at different recovery stages. In addition, patients can actively exert horizontal pushing force on the end effector, enabling quantitative assessment of muscle strength and partially replacing traditional subjective evaluations that rely on expert experience.

## 3 Evaluation methods

### 3.1 Evaluation method

The upper-limb rehabilitation status of stroke patients fundamentally reflects their overall motor ability during task-oriented movements, which depends not only on joint range of motion (ROM) but also on movement control quality and voluntary force output (Macedo and Magee, 2008). Therefore, a comprehensive rehabilitation assessment should simultaneously capture movement outcomes, movement process characteristics, and dynamic capabilities.

Traditional ROM measurements reflect only the static extrema of individual joints and are insufficient to characterize functional movements under multi-joint coordination (Murphy et al., 2010). In horizontal-plane upper-limb robot-assisted training, ROM is difficult to measure accurately due to mechanical and sensor constraints and cannot be directly mapped to task performance. To address this limitation, TO is adopted as an indirect metric. By comparing the actual movement trajectory with the reference trajectory, TO reflects the upper bound of multi-joint mobility and task execution capability.

Movement process quality is another critical component of upper-limb function. Stroke patients often exhibit uneven velocity, frequent pauses, and trajectory corrections during movement, indicating impaired motor control. Therefore, MS is selected as the second feature to evaluate movement continuity and coordination, complementing TO, which primarily reflects movement outcomes.

From the dynamic perspective, voluntary force output determines whether patients can transition from passive or assisted training to active training. Rehabilitation robots can measure interaction forces between the patient and the robot in real time. HPF is used to represent muscle strength, reflecting the patient’s voluntary output capacity along the task direction.

In summary, considering the characteristics of robot-assisted training and task-oriented rehabilitation, TO, MS, and HPF are selected as the three key features representing upper-limb rehabilitation status in stroke patients, and they serve as inputs to the subsequent rehabilitation assessment model.

### 3.2 Feature extraction and processing

TO is used to quantitatively evaluate the spatial matching between the patient’s actual movement trajectory and the intended reference trajectory. Methods for assessing TO can be based on shape, point sets, or line segments. In this study, the Hausdorff distance based on shape is employed to compute TO. Essentially, the Hausdorff distance measures the maximum of minimum distances between two point sets and is sensitive to outlier points (Huttenlocher et al., 1993). To ensure trajectory integrity while reflecting the influence of outliers, a weighted mean distance is adopted to improve the Hausdorff method.Let the reference trajectory be represented by the point set $A=\left\{a_{1},a_{2},\ldots ,a_{m}\right\}$ and $B=\left\{b_{1},b_{2},\ldots ,b_{n}\right\}$ , where $a_{i\;}$ and $b_{j\;}$ denote two-dimensional sampled points, and m and n are the numbers of sampled points in the reference and actual trajectories, respectively. The formula for trajectory overlap is presented in Equation 6:

$$TO=\frac{1}{2}\left(\frac{1}{m}\sum_{m}^{i=1}\max\left(0,1-\frac{\min_{b\in B}∥a_{i}-b∥}{\delta }\right)+\frac{1}{n}\sum_{n}^{j=1}\max\left(0,1-\frac{\min_{a\in A}∥b_{j}-a∥}{\delta }\right)\right)$$ (6)

where, $∥a_{i}-b∥$ denotes the Euclidean distance between the reference point $a_{i}$ and the actual point *b*, and δ the maximum clinically acceptable trajectory deviation threshold, defined by rehabilitation experts according to the difficulty level of the training task. When $∥a_{i}-b∥\leq \delta$ , the matching score increases linearly with decreasing distance. Otherwise, when $∥a_{i}-b∥>\delta$ , the matching score is assigned a value of 0. In this study, δ is set to 15 mm based on clinical consensus and repeated preliminary tests, ensuring both tolerance for physiological tremor and sensitivity to motor impairment.

A higher TO value indicates greater trajectory consistency, whereas lower values reflect larger deviations, capturing the influence of local outliers. TO ranges from 0 to 1, with values closer to one corresponding to high trajectory overlap and better movement control, and values closer to 0 indicating substantial deviation and reduced movement quality. This method accounts for both global trajectory consistency and local errors, enabling an objective quantification of movement quality during rehabilitation.

MS is used to characterize the continuity and stability of the patient’s movement process (Rohrer et al., 2002). Based on the collected position information of the end-effector, the trajectory signal is differentiated with respect to time to sequentially obtain the velocity and acceleration. The acceleration signal is further differentiated with respect to time to derive the jerk signal, and MS expressed in Equation 7:

$$J\left(t\right)=\frac{d^{3}x\left(t\right)}{dt^{3}}$$ (7)

Since jerk is highly sensitive to abrupt changes and oscillations during movement, it can effectively reflect motion abnormalities caused by unstable neuromuscular control and has therefore been widely used in movement quality analysis in the rehabilitation field. To improve noise robustness and ensure numerical stability, the raw kinematic data are preprocessed using a low-pass filter before jerk calculation. To comprehensively evaluate the smoothness of the entire movement process, the MSE of the filtered and normalized jerk signal is adopted as the MS feature, and Equation 8 is obtained accordingly:

$$MS=\frac{1}{N}\sum_{i=1}^{N}J_{i}^{2}$$ (8)

where N denotes the number of trajectory sample points and $J_{i}$ represents the jerk value at the *i*th sample point. A smaller MS value indicates a smoother movement process, reflecting better motor control and coordination in the patient.

In the force-measurement training mode, the rehabilitation robot acquires the patient’s horizontal pushing force through a force sensor. To improve reliability, the average value over ten consecutive repetitions is adopted as the HPF feature. Meanwhile, the variability and repeatability are analyzed using the coefficient of variation (CV) and intraclass correlation coefficient (ICC). The results show that CV < 8% and ICC >0.85 for all subjects, indicating that the ten-repetition average can effectively reduce random fluctuations and ensure good stability and reproducibility of HPF.

### 3.3 Establishment of BRB rehabilitation evaluation model

To scientifically evaluate the upper-limb motor function rehabilitation status of patients with stroke, the nonlinear relationship between the rehabilitation status and the health-related feature variables is first established as follows:

$$H=f\left(x_{1t},x_{2t},\cdots ,x_{Mt},V\right)$$

where, H denotes the upper-limb motor function health status of the patient; x denotes the health-related feature variables; f denotes the nonlinear relationship; and V denotes the model parameters. In this study, based on semi-quantitative information, BRB theory is employed to establish the nonlinear relationship f between the feature variables and the motor function assessment model using key features such as TO, MS during motion, and HPF.

For the BRB-based upper-limb motor function assessment model, interpretable health-related feature variables are first selected and constructed from the raw data by incorporating expert knowledge, and these features are used as inputs to the BRB. The rehabilitation assessment results are then obtained using the ER method. Subsequently, to improve the accuracy and reliability of the model, a parameter optimization framework is established, in which the BRB parameters are iteratively optimized using the differential evolution (DE) algorithm. In this way, a simple yet accurate assessment model for individual upper-limb rehabilitation status is developed. The specific modeling procedure is described as follows:

Step 1: Model construction. A basic BRB consists of a set of simple IF–THEN rules derived from expert knowledge, in which expert-assigned belief degrees are used to establish the mapping between the inputs and the output. The *k*th rule for rehabilitation assessment is described as follows:

$$\begin{array}{rr} & R_{k}:\;If\;x_{1}\;is\;A_{1}^{k}\wedge x_{2}\;is\;A_{2}^{k}\wedge x_{M}\;is\;A_{M}^{k}\;Then\left(\left(D_{1},\beta_{1,k}\right),\left(D_{2},\beta_{2,k}\right),...,\left(D_{N,k}\right)\right)\\ & with\;a\;rule\;weight\theta_{k}\;and\;attribute\;weight{\;\delta }_{1},\delta_{2},...,\delta_{M} \end{array}$$

where, $R_{k}$ denotes the *k*th confidence rule; $x_{i\;}$ denotes the *i*th premises attribute, *i* represents the number of feature quantities of system, i = 1, 2, … ,M; $A_{i}^{k}$ denotes the reference value of the *i*th premises attribute in the *k*th rule, i = 1, 2, … , k = 1, 2, … , L; $M_{k}$ denotes the number of prerequisite attributes in *k*th rule; $D_{j}$ denotes the *j*th evaluation result, j = 1, 2, … , N; $\beta_{j,k}$ denotes the confidence of the *k*th result $D_{j}$ , j = 1, 2, … , N, k = 1, 2, … , L; If there are M premise attributes in the BRB, then

$$\delta_{i}=\delta_{i,k},\bar{\delta_{i}}=\frac{\delta_{i}}{\max_{i=1,2,...,M}\left\{\delta_{i}\right\}},\left(i=1,2,\cdots ,M,k=1,2,...,L\right).$$

If $\sum_{j=1}^{N}\beta_{j,k}\neq 1,$ the *k*th rule is considered incomplete; otherwise, it is complete.

**FIGURE 9:**
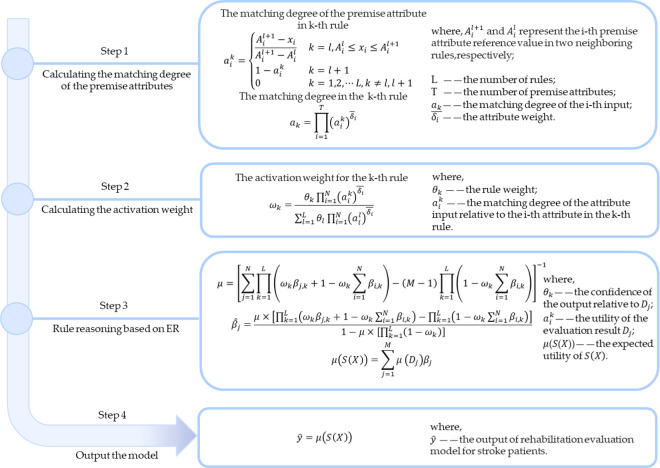
Reasoning process based on ER.

Step 2: Model inference. To obtain the final system output, the belief rules are aggregated using the ER algorithm. Figure 9 depicts the detailed inference procedure.
Step 3: Model Optimization. In the BRB-based upper-limb rehabilitation assessment model for stroke patients, the initial model parameters are usually determined by domain experts based on their experience and knowledge. However, expert knowledge is inherently subjective and varies among individuals, which may lead to inconsistency and uncertainty in the model parameters and consequently affect the accuracy and reliability of the BRB model.Therefore, optimization algorithms can be employed to adjust and optimize the model parameters. Compared with particle swarm optimization and genetic algorithms, DE requires fewer parameters to be tuned, exhibits strong global search capability, and is less likely to be trapped in local optima (Islam et al., 2020). As a result, DE can provide more accurate and reliable evaluation results for rehabilitation specialists.

During the optimization process of the BRB-based rehabilitation assessment model, Equation 9 is obtained as the objective function:

$$\begin{array}{cc} & \min\xi \left(V\right)\\ & 0\leq \theta_{k}\leq 1\\ & 0\leq \beta_{j,k}\leq 1,j=1,..,N,k=1,2,\ldots L\\ & \sum_{n=1}^{N}\beta_{j,k}\leq 1\\ & 0\leq \delta_{i}\leq 1,i=1,\ldots ,M \end{array}$$ (9)

where $\xi \left(V\right)$ represents the MSE, and Equation 10 is obtained accordingly:

$$\xi \left(V\right)=\frac{1}{T}\sum_{n=1}^{T}{\left(y_{n}-{\hat{y}}_{n}\right)}^{2}$$ (10)

where, $V={\left[\theta_{k},\delta_{i},\beta_{j,k}\right]}^{T\;}$ denotes the set of parameters to be optimized; $y_{n}$ denotes the actual rehabilitation state of stroke patients; ${\hat{y}}_{n}$ denotes the output of the rehabilitation assessment model; and T denotes the number of samples. T denotes the amount of data.

## 4 Case study

To validate the effectiveness and accuracy of the proposed upper-limb motor function assessment model for post-stroke patients, a 5-bar upper-limb rehabilitation robot was employed to conduct objective quantitative evaluations during active training. Upper-limb position and force data were collected in real time through an integrated motion control card and processed to extract key health-related feature parameters reflecting rehabilitation status. Participants performed circular, rectangular, and triangular trajectory-tracking tasks in active mode. TO was calculated by comparing the actual trajectories with predefined standard templates to assess spatial motor control. Since trajectory coincidence mainly reflects geometric similarity and cannot capture velocity variation or movement continuity, MS was further introduced to evaluate movement rhythm and coordination. In addition, the mean value of ten consecutive horizontal pushing forces was calculated to assess upper-limb muscle strength, endurance, and force stability. The results showed that healthy subjects exhibited stable HPF, whereas post-stroke patients presented significant fluctuations due to neurological impairments, indicating that horizontal force can serve as an objective indicator for rehabilitation assessment and training design. The duration of data acquisition for each trajectory task was controlled within 5–10 min to ensure reliability and statistical validity. In total, motion data from 236 post-stroke patients at different rehabilitation stages and 120 healthy subjects were collected for model construction and validation. All data were obtained with informed consent from the participants in accordance with relevant ethical requirements. Table 1 presents the baseline demographic and clinical characteristics of the stroke patients. The cohort consisted of 83.9% males and 16.1% females, with a mean age of 60.3 ± 11.8 years. Regarding stroke etiology, 86.4% of patients had ischemic stroke and 13.6% had hemorrhagic stroke. In terms of the affected side, 29% of patients presented with left-side impairment, while 71% had right-side impairment. The baseline upper-limb motor function was assessed using the Fugl–Meyer Assessment for Upper Extremity (FMA-UE), with an average score of 41.2 ± 15.7 (range: 0–66). These characteristics provide a comprehensive description of the study population and ensure the representativeness and interpretability of the experimental results.

**TABLE 1:** Baseline demographic and clinical characteristics of stroke patients (N = 236).

Measure	Sex (male/Female)	Age (year)	Etiology (ischemic/Hemorrhagic)	Side (left/Right)	FMA-UE score (total)
Value	83.9% / 16.1%	60.3 ± 11.8	86.4% / 13.6%	29% / 71%	41.2 ± 15.7

**FIGURE 10:**
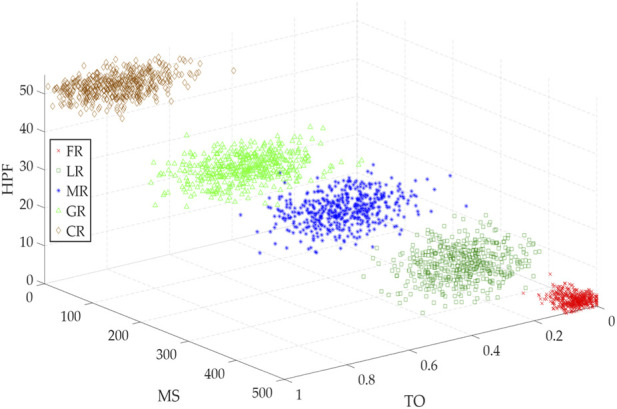
Experimental data of five levels of upper-limb motor function status in stroke patients.

In clinical practice, the Fugl–Meyer Assessment, the Ueda Sensory Assessment, and the Motor Status Scale are widely used to evaluate upper-limb motor function in stroke patients; however, these scales primarily focus on joint range of motion, postural control, and task performance, and are limited in their ability to directly and objectively quantify muscle strength, endurance, and force control. In contrast, upper-limb rehabilitation robots can provide controllable external loading and high-precision position and force feedback, enabling the synchronized acquisition of dynamic information such as motion trajectories, mechanical characteristics, and movement quality. Furthermore, traditional clinical scales are not directly applicable to the rehabilitation robot used in this study. Therefore, three rehabilitation experts were invited to develop a customized assessment scale, among whom one had more than 10 years of clinical experience. Based on patients’ performance during robot-assisted training, the experts established an evaluation framework tailored to the system. The expert evaluation was conducted in a double-blinded manner: each expert was blinded to the assessment results of the other two specialists and completely blinded to the kinematic and force data collected by the rehabilitation robot, ensuring independent and unbiased judgment. By integrating expert knowledge with quantitative indicators, including trajectory overlap, movement smoothness, and horizontal pushing force, a more comprehensive and objective assessment framework was constructed, as detailed in Table 2. Inter-rater reliability was verified using the intraclass correlation coefficient (ICC), with ICC = 0.82 (>0.75), indicating excellent consistency among the experts. During the training process, rehabilitation experts evaluated each patient’s rehabilitation level according to this scale, and the motion data of all participants obtained through this assessment are illustrated in Figure 10, providing a reliable and objective basis for subsequent feature analysis and model development.

**TABLE 2:** The levels of upper limb motor function recovery.

Recovery level	Description
Complete recovery (CR)	Patient performs upper-limb movements accurately, with normal coordination and muscle strength
Good recovery (GR)	Patient’s upper-limb function is largely restored, enabling most daily tasks, with some limitations in fast or complex movements
Moderate recovery (MR)	Patient can perform basic upper-limb movements but shows noticeable deficits in coordination and force output
Limited recovery (LR)	Patient can perform only limited movements, with evident deficiencies in movement control and unstable force output
Functionally restricted (FR)	Patient is unable to perform normal upper-limb movements independently, exhibiting severe motor dysfunction

During the construction of the BRB model, to avoid the exponential growth in the number of rules caused by an excessive number of feature reference values, each input feature was uniformly divided into three reference levels based on expert knowledge. This setting ensures that the model retains sufficient discriminative capability while keeping the number of rules within a reasonable range, thereby effectively reducing computational complexity and the difficulty of parameter optimization. At the same time, the three-level partitioning is consistent with common clinical cognition and enables the model to maintain stability and reliability under limited sample conditions. As a result, a reasonable balance is achieved between model expressiveness and computational efficiency.

For TO, it quantifies how closely the patient’s trajectory aligns with the target trajectory:

$$A_{1}^{k}\in \left\{\text{Low }\left(L\right),\text{Medium }\left(M\right),\text{High }\left(H\right)\right\}$$

For MS, it assesses the coordination and stability of the movement:

$$A_{2}^{k}\in \left\{\text{Poor }\left(P\right),\text{Moderate }\left(M\right),\text{Good }\left(G\right)\right\}$$

For HPF, it measures the patient’s force output during upper limb active movement:w

$$A_{3}^{k}\in \left\{\text{Weak }\left(W\right),\text{Medium }\left(M\right),\text{Strong }\left(S\right)\right\}$$

For the evaluation results, as shown in Table 2, there are five health states, denoted as FR, LR, MR, GR, CR, that is: {Q1, Q2, Q3, Q4, Q5} = {FR,LR,MR,GR,CR}.

Based on expert experience and the characteristics of the sampled data shown in Figure 10, the semantic reference values of TO, MS, HPF, and the evaluation results were quantified, as shown in Tables 3–6.

**TABLE 3:** The attribute reference value of TO.

Referential points	L	M	H
Referential values	0.2	0.4	0.8

**TABLE 4:** The attribute reference value of MS.

Referential points	P	M	G
Referential values	450	350	150

**TABLE 5:** The attribute reference value of HPF.

Referential points	W	M	S
Referential values	10	20	40

**TABLE 6:** The reference value of rehabilitation status.

Referential points	FR	LR	MR	GR	CR
Referential values	1	2	3	4	5

In the rehabilitation assessment model, since the BRB contains three input features and each feature has three reference values, a total of 27 belief rules can be constructed to comprehensively evaluate the upper-limb motor function status of stroke patients. The corresponding initial parameters are listed in Table 7.

**TABLE 7:** The initial parameters of BRB.

Rule number	Attributes			Upper limb motor function assessment results{Q_1_,Q_2_,Q_3_,Q_4_,Q_5_} = {1,2,3,4,5}	Rule weights
	TO	MS	HPF		
	1	1	1		
1	L	P	W	{(1,0,0,0,0)}	1
2	M	P	W	{(0.6,0.1,0,0.1,0.1)}	1
3	H	P	W	{(0.6,0.4,0,0,0)}	1
4	L	M	W	{(0.4,0,0.3,0.3,0)}	1
5	M	M	W	{(0.6,0,0.3,0,0)}	1
6	H	M	W	{(0.3,0.3,0.2,0.2,0)}	1
7	L	G	W	{(0.2,0,0,0.1,0.6)}	1
8	M	G	W	{(0.1,0.4,0.1,0.4,0)}	1
9	H	G	W	{(0.3,0.1,0,0.3,0.3)}	1
10	L	P	M	{(0,0,0.7,0.3,0)}	1
11	M	P	M	{(0.2,0.2,0.2,0.2,0.2)}	1
12	H	P	M	{(0.2,0.1,0.3,0.3,0)}	1
13	L	M	M	{(0,0.3,0.1,0,0.5)}	1
14	M	M	M	{(0,0.3,0.3,0.1,0.3)}	1
15	H	M	M	{(0.1,0.3,0.1,0.2,0.3)}	1
16	L	G	M	{(0.5,0.4,0,0.1,0)}	1
17	M	G	M	{(0.1,0,0.3,0.6,0)}	1
18	H	G	M	{(0,0,0,0.7,0.3)}	1
19	L	P	S	{(0.4,0.2,0.1,0.3,0)}	1
20	M	P	S	{(0,0.3,0,0.4,0.2)}	1
21	H	P	S	{(0.3,0.2,0.2,0.3,0)}	1
22	L	M	S	{(0.2,0.3,0.1,0.3,0.1)}	1
23	M	M	S	{(0.3,0.1,0,0.3,0.3)}	1
24	H	M	S	{(0,0.1,0.3,0.2,0.4)}	1
25	L	G	S	{(0,0.3,0.5,0,0.1)}	1
26	M	G	S	{(0.1,0,0.3,0.5,0)}	1
27	H	G	S	{(0,0,0,0.4,0.6)}	1

**FIGURE 11:**
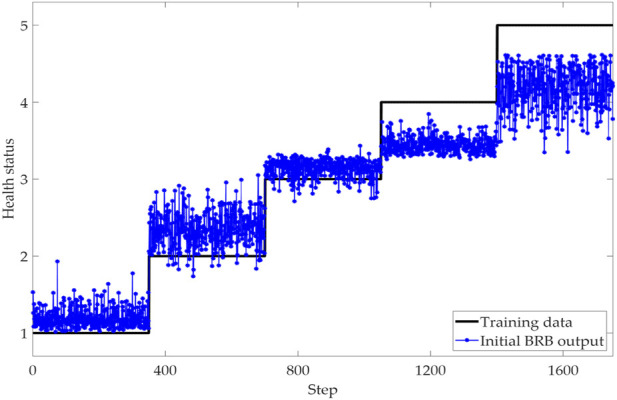
Health assessment results obtained using the initial BRB.

A total of 2,500 valid data samples were collected from 356 subjects. To eliminate data leakage and ensure reliable model validation, a strict subject-level stratified random split was implemented. All data from each individual subject were assigned entirely to either the training set or the test set, preventing any overlap of patient data between the two subsets. In this way, 1,750 samples were used for model training and parameter optimization, while 750 independent samples were used for testing, thereby ensuring that the model evaluation is fair, unbiased, and representative, while also preserving the diversity of the data for a comprehensive evaluation of the BRB model on unseen samples. Based on the initial parameters provided by experts, feature weights were not taken into account; both parameters $\theta_{k\;}$ and $\delta_{i}$ were set to 1, implying that each rule and each feature were considered equally important. The belief degrees associated with each evaluation outcome for every rule were determined according to expert knowledge, thereby yielding the preliminary health assessment results. Figure 11 indicates that the assessment results exhibit relatively low fitting accuracy with the training data.

**FIGURE 12:**
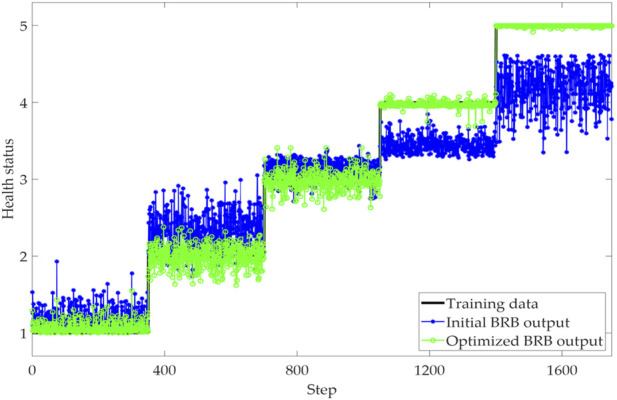
Health assessment results obtained using the DE-optimized BRB.

To reduce the subjectivity introduced by expert knowledge and obtain a more accurate assessment of upper-limb rehabilitation status, the parameters of the initial BRB model were optimized using DE. During the optimization process, the control parameters F *,* CR , and NP were set to 0.5, 0.8, and 1000, respectively. These values were selected based on commonly adopted empirical settings in the literature and were found to provide a good balance between global exploration and convergence stability for the BRB parameter optimization problem. The updated parameters are listed in Table 8, and the assessment results on the training data are shown in Figure 12. Compared with the initial BRB model, the optimized model exhibits significantly improved output performance and achieves a better fit to the training data.

**TABLE 8:** The optimized BRB parameters.

Rule number	Attributes			Upper limb motor function assessment results{Q_1_,Q_2_,Q_3_,Q_4_,Q_5_} = {1,2,3,4,5}	Rule weights
	TO	MS	HPF		
	0.3791	0.6284	0.4734		
1	L	P	W	{(0.9995,0.0001,0.0001,0,0.0004)}	0.0463
2	M	P	W	{(0.6304,0.062,0.0059,0.2836,0.0181)}	0
3	H	P	W	{(0.3858,0.5811,0.0067,0.0262,0.0001)}	0.9775
4	L	M	W	{(0.0101,0.2338,0.3498,0.3726,0.0337)}	0
5	M	M	W	{(0.0025,0.9942,0.0023,0.0008,0.0002)}	0.0645
6	H	M	W	{(0.0102,0.1477,0.7773,0.0018,0.063)}	0.9168
7	L	G	W	{(0.0361,0.4046,0.0192,0.0079,0.5322)}	0.6023
8	M	G	W	{(0.0852,0.3398,0.0093,0.0644,0.5013)}	0.001
9	H	G	W	{(0.0525,0.8977,0.0174,0.0039,0.0284)}	0.0023
10	L	P	M	{(0.0003,0.8666,0.0777,0.003,0.0524)}	0.0001
11	M	P	M	{(0.0064,0.5634,0.3544,0.0512,0.0246)}	0.0485
12	H	P	M	{(0.1143,0.006,0.4608,0.4097,0.0092)}	0.7953
13	L	M	M	{(0.7477,0.0101,0.1077,0.0719,0.0625)}	0.0299
14	M	M	M	{(0.0252,0.0137,0.0867,0.7172,0.1572)}	0.0294
15	H	M	M	{(0.7979,0.0015,0.0341,0.164,0.0025)}	0.0021
16	L	G	M	{(0.1495,0.206,0.0631,0.3172,0.2642)}	0.0594
17	M	G	M	{(0.0399,0.1201,0.1146,0.3878,0.3376)}	0.0001
18	H	G	M	{(0.3316,0.1085,0.0361,0.3376,0.1862)}	0.0001
19	L	P	S	{(0.9172,0.0332,0.0009,0.0035,0.0452)}	0.8431
20	M	P	S	{(0.3517,0.0049,0.0007,0.6183,0.0244)}	0.7856
21	H	P	S	{(0.0128,0.022,0.0213,0.9402,0.0037)}	0.9015
22	L	M	S	{(0.0996,0.038,0.0492,0.008,0.8052)}	0.2483
23	M	M	S	{(0.0083,0.05,0.6035,0.0114,0.3268)}	0.0002
24	H	M	S	{(0.16,0.0014,0.4507,0.0074,0.3805)}	0
25	L	G	S	{(0.1855,0.2524,0.4585,0.1036,0)}	0.0012
26	M	G	S	{(0.6818,0.1755,0.0052,0.1367,0.0008)}	0
27	H	G	S	{(0,0.0001,0.0002,0,0.9997)}	0.0237

**FIGURE 13:**
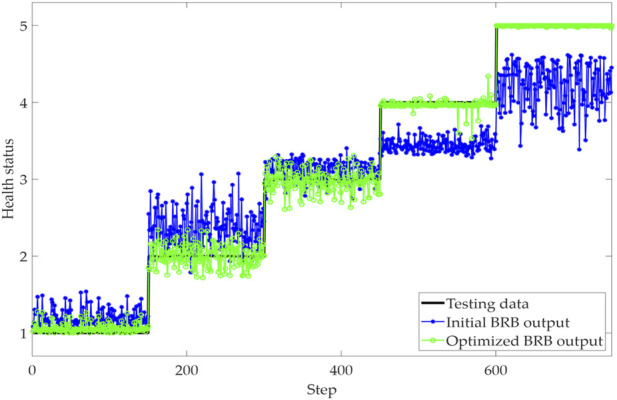
Health assessment results obtained using the DE-optimized BRB for the testing data.

To further assess the generalization and stability of the model, the test dataset was input into the BRB model with the DE-optimized parameters unchanged, The corresponding outputs are presented in Figure 13. The results show that the MSE for both the training and test data remains relatively low, indicating strong agreement between model predictions and actual rehabilitation assessment values. These findings demonstrate that the proposed model exhibits robust generalization performance and stability on unseen data.

## 5 Comparative analysis

To validate the accuracy and practicality of the proposed upper-limb motor function assessment method, BP and SVM were selected as representative machine learning models for comparison. BP, with its multilayer structure and error backpropagation mechanism, has been widely applied to nonlinear problems such as pattern recognition, diagnosis, and prediction (Rumelhart et al., 1986). SVM constructs an optimal separating hyperplane and demonstrates strong stability and generalization capability in small-sample, nonlinear classification and regression tasks. Therefore, the selection of these two models allows for a comprehensive evaluation of the performance advantages of the proposed BRB model under different modeling mechanisms (Cortes and Vapnik, 1995).

**FIGURE 14:**
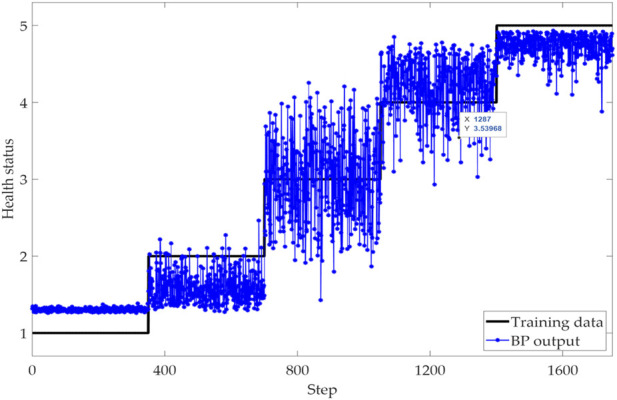
Health assessment results obtained using the BP neural network.

**FIGURE 15:**
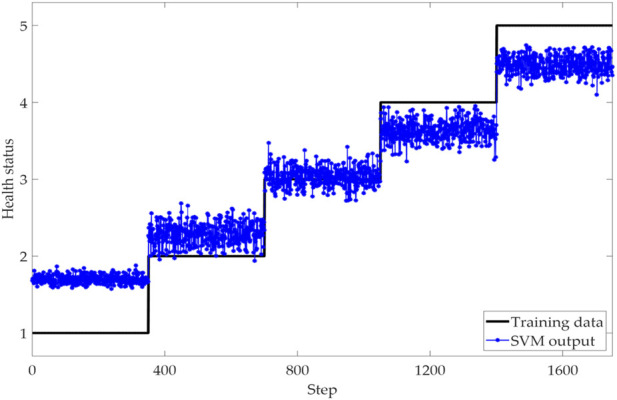
Health assessment results obtained using the SVM neural network.

For the upper-limb motor function rehabilitation assessment of stroke patients, all three models employed the same dataset for training and testing to ensure fairness and consistency in the comparison. Specifically, 1750 samples were used as training data for parameter learning and optimization, and the remaining 750 samples were used as test data to evaluate model generalization performance. The assessment results obtained using BP are shown in Figure 14. Although BP can capture the general trend of upper-limb motor function changes to some extent, noticeable prediction deviations are observed in certain sample intervals, indicating limited stability in small-sample and highly uncertain data environments. To further evaluate the practicality and robustness of the proposed assessment model, SVM was applied to the same dataset, and the corresponding results are presented in Figure 15. Compared with BP, SVM provides improved prediction stability; but it still struggles to accurately characterize gradual transitions among different motor function levels due to the inherent fuzziness and uncertainty in rehabilitation assessment.

Table 9 presents a comprehensive comparison of the performance of the proposed BRB model, the BP neural network, and the SVM model for upper-limb motor function rehabilitation assessment in stroke patients. The evaluation is conducted using six key metrics, including MAE, MBE, MAPE, MSE, RMSE, and the coefficient of determination *R* ^2^. Across both the training and test sets, the semi-quantitative information-based BRB model consistently outperforms the BP and SVM models on all evaluation metrics. Specifically, the BRB model achieves the lowest values in MAE, MBE, MAPE, MSE, and RMSE, together with the highest *R* ^2^ values, indicating not only superior fitting performance on the training data but also excellent generalization capability on unseen test data. In particular, the BRB model attains a remarkably low MSE of 0.011 on the test set, which is substantially lower than that of the BP model and the SVM model. Meanwhile, its test-set *R* ^2^ reaches 0.9945, significantly exceeding the performance of the two comparison models. These findings demonstrate that the proposed BRB model provides superior assessment accuracy, enhanced data-fitting capability, and stronger stability and generalization performance. Therefore, it is more suitable for addressing the complexity and uncertainty inherent in clinical stroke upper-limb rehabilitation evaluation.

**TABLE 9:** Comparison of rehabilitation assessment performance on training and test sets.

Models	MAE		MBE		MAPE		MSE		RMSE		*R* ^2^	
	Train	Test	Train	Test	Train	Test	Train	Test	Train	Test	Train	Test
BP	0.3602	0.3525	−0.0363	−0.0256	0.1612	0.1581	0.1756	0.1713	0.419	0.4139	0.9122	0.9143
SVM	0.3935	0.4008	0.0287	0.0333	0.2143	0.2164	0.2076	0.2094	0.4557	0.4576	0.8962	0.8953
BRB	0.0638	0.068	0.0025	0.0018	0.0338	0.0345	0.0098	0.011	0.099	0.1048	0.9951	0.9945

## 6 Discussion

This study was motivated by the high subjectivity, low efficiency, and poor consistency of traditional clinical scales for stroke upper-limb rehabilitation assessment, as well as the limited interpretability and clinical integration of most data-driven robot-assisted methods. To address these issues, a horizontal upper-limb rehabilitation robot was developed, and a semi-quantitative BRB-based assessment model was proposed. This section evaluates the proposed method in terms of modeling mechanism, data adaptability, and clinical interpretability, through comparison with two representative data-driven approaches: BP and SVM.

BP is a classical feedforward neural network trained via error backpropagation, widely used for nonlinear approximation and pattern recognition. Although it exhibits strong nonlinear mapping capability through multilayer structures, its application in rehabilitation assessment is constrained by its black-box nature, which prevents incorporation of clinical prior knowledge and limits interpretability in decision-making. Moreover, BP is highly sensitive to data quality and sample size; the small-scale, noisy, and heterogeneous nature of rehabilitation datasets often leads to overfitting and unstable generalization. In addition, its iterative optimization process depends strongly on parameter initialization and may converge to suboptimal solutions. These factors collectively result in inferior predictive performance, as reflected by higher MAE, MSE, RMSE, and lower *R* ^2^ on both training and test sets. SVM, grounded in statistical learning theory, enhances generalization by constructing an optimal separating hyperplane in a high-dimensional feature space. It generally demonstrates better performance than BP under small-sample conditions and improved robustness to noise. However, SVM remains fundamentally a data-driven model and lacks the capability to incorporate qualitative clinical reasoning. In addition, it is limited in modeling complex nonlinear interactions among multi-dimensional motion features and struggles to capture the gradual and fuzzy transitions between rehabilitation stages. Its performance is also highly dependent on kernel selection and hyperparameter tuning, which reduces robustness across heterogeneous datasets. Consequently, although SVM improves generalization compared with BP, it still fails to satisfy the accuracy and interpretability requirements of clinical rehabilitation assessment. In contrast, the proposed BRB framework integrates expert knowledge and data-driven learning through a belief rule base structure, enabling explicit representation of clinical reasoning. The evidential reasoning nference mechanism effectively handles uncertainty arising from noise, incompleteness, and variability in rehabilitation data. Furthermore, differential evolution optimization reduces reliance on subjective expert tuning and improves parameter consistency. Through this hybrid mechanism, the BRB model achieves superior predictive accuracy and stability compared with BP and SVM, while maintaining transparent and interpretable decision outputs, which is critical for clinical application. From a clinical perspective, the proposed system establishes a unified framework linking robotic sensing with functional assessment. The horizontal 5-bar rehabilitation robot enables stable acquisition of multi-dimensional kinematic and force features, while the BRB model transforms these heterogeneous signals into interpretable rehabilitation levels. This integration reduces clinician workload, improves evaluation consistency, and supports personalized rehabilitation planning. Unlike BP and SVM, which function as opaque prediction models, the proposed framework provides a transparent reasoning process aligned with clinical decision logic.

Despite its advantages, the performance of the proposed system is still constrained by the limitations of the current robotic platform. The horizontal 5-bar mechanism restricts motion to planar trajectories, limiting the diversity of captured movement patterns. However, activities of daily living typically involve complex three-dimensional multi-joint coordination, particularly vertical movements against gravity. This limitation may reduce the completeness of functional representation in more complex rehabilitation scenarios. Future work will therefore focus on extending the robotic system to higher degrees of freedom and improving the flexibility of BRB parameter learning, to enable more comprehensive and realistic rehabilitation assessment.

## 7 Conclusion

This study proposes a BRB-based semi-quantitative assessment framework for upper-limb motor function in stroke patients, addressing the strong subjectivity of traditional clinical scale evaluations and the lack of objective quantitative support. It also overcomes the limitations of existing BRB methods, such as insufficient use of real rehabilitation robot data, incomplete multi-dimensional motor performance characterization, and the absence of a unified closed-loop assessment system. A two-DOF horizontal upper-limb rehabilitation robot was developed. A BRB assessment model was constructed by integrating robot-collected motion data and clinical expert knowledge. Three representative features—trajectory overlap, movement smoothness, and horizontal pushing force—were selected to characterize upper-limb motor performance. The ER approach was used to fuse heterogeneous and uncertain multi-source information. The DE algorithm optimized model parameters to improve robustness and stability. Experimental results show that the proposed method can evaluate upper-limb motor function in stroke patients effectively and consistently. Compared with BP and SVM models, the proposed framework achieves superior accuracy, fitting ability, and generalization capability, validating the effectiveness of combining BRB and ER for uncertainty-aware clinical assessment. Future work will extend the rehabilitation robot to three-dimensional motion by adding a vertical degree of freedom. Large-scale multi-center clinical studies will be conducted to further verify the robustness and clinical generalizability of the method. More comprehensive kinematic, dynamic, and physiological indicators will be incorporated to improve the precision and interpretability of rehabilitation evaluation, supporting the development of personalized closed-loop intelligent rehabilitation systems.

## Data Availability

The data analyzed in this study is subject to the following licenses/restrictions: The dataset presented in this paper contains sensitive patient information protected by hospital privacy policies and is not publicly available. Requests for access may be directed to the second author for further discussion. Requests to access these datasets should be directed to WS, swcwork2026@163.com.
